# Humanized Tau Mice with Regionalized Amyloid Exhibit Behavioral Deficits but No Pathological Interaction

**DOI:** 10.1371/journal.pone.0153724

**Published:** 2016-04-12

**Authors:** Michael J. Yetman, Stephanie W. Fowler, Joanna L. Jankowsky

**Affiliations:** 1 Department of Neuroscience, Baylor College of Medicine, Houston, Texas, United States of America; 2 Department of Neurology, Baylor College of Medicine, Houston, Texas, United States of America; 3 Department of Neurosurgery, Baylor College of Medicine, Houston, Texas, United States of America; Nathan Kline Institute and New York University Langone Medical Center, UNITED STATES

## Abstract

Alzheimer’s disease (AD) researchers have struggled for decades to draw a causal link between extracellular Aβ aggregation and intraneuronal accumulation of microtubule-associated protein tau. The amyloid cascade hypothesis posits that Aβ deposition promotes tau hyperphosphorylation, tangle formation, cell loss, vascular damage, and dementia. While the genetics of familial AD and the pathological staging of sporadic disease support this sequence of events, attempts to examine the molecular mechanism in transgenic animal models have largely relied on models of other inherited tauopathies as the basis for testing the interaction with Aβ. In an effort to more accurately model the relationship between Aβ and wild-type tau in AD, we intercrossed mice that overproduce human Aβ with a tau substitution model in which all 6 isoforms of the human protein are expressed in animals lacking murine tau. We selected an amyloid model in which pathology was biased towards the entorhinal region so that we could further examine whether the anticipated changes in tau phosphorylation occurred at the site of Aβ deposition or in synaptically connected regions. We found that Aβ and tau had independent effects on locomotion, learning, and memory, but found no behavioral evidence for an interaction between the two transgenes. Moreover, we saw no indication of amyloid-induced changes in the phosphorylation or aggregation of human tau either within the entorhinal area or elsewhere. These findings suggest that robust amyloid pathology within the medial temporal lobe has little effect on the metabolism of wild type human tau in this model.

## Introduction

Alzheimer’s disease (AD) has classically been defined as an age-dependent neurodegenerative dementia in which the interplay of aberrant intra-neuronal and extracellular protein aggregates results in a complex set of mnemonic and cognitive symptoms. For decades, the prevailing theory of AD etiology has been the amyloid cascade hypothesis, which posits that intra-neuronal formation of neurofibrillary tangles and subsequent cellular degeneration are initiated by the aggregation of amyloid-β (Aβ) into extracellular plaques [1, 2]. While human genetic data and preclinical animal studies provide evidence that the production of Aβ plays a critical role in pathogenesis, the absence of neurofibrillary tangles and appreciable neurodegeneration in models of Alzhiemer’s amyloidosis remain significant challenges for the field [3].

In an effort to create animal models bearing neurofibrillary tangles, many groups have turned to tau mutations responsible for frontotemporal lobar degeneration (FTLD) [4, 5]. Nearly two dozen lines of transgenic mice overexpressing mutant human tau have been described (http://www.alzforum.org/research-models). In addition to the accumulation of insoluble hyper-phosphorylated tau, several of these transgenic lines also develop frank neuronal loss that has eluded models of CNS amyloidosis [6–8]. Mice bearing FTLD tau variants were instrumental in probing the relationship between Aβ and tau in vivo, revealing that tau hyperphosphorylation could be accelerated by the introduction of Aβ either through intracranial injection or transgenic expression [9–13].

While past experiments demonstrated a clear pathogenic relationship between Aβ and tau, most of these studies relied on models bearing a variant form of tau to enhance its propensity for aggregation. In reality, these variants have never appeared in familial AD. Here we set out to test whether Aβ could exacerbate hyperphosphorylation of wild-type tau in the same manner as had been shown for FTLD variants. We employed a tau transgenic model created by Andorfer et al. to express wild-type human tau in the absence of mouse tau [14–16]. This model uses the human tau promoter to maintain the normal spatiotemporal pattern of expression, and produces all six human tau isoforms within the mouse brain. We introduced human Aβ into this htau model using a second transgenic line carrying a chimeric mutant version of the amyloid precursor protein (APP) [17]. To explore the spatial relationship between amyloid pathology and tau hyperphosphorylation, we initiated Aβ deposition in the entorhinal cortex using the neuropsin promoter [18, 19]. This compound model allowed us to test whether Aβ accumulation was capable of exacerbating the pathological phosphorylation of wild-type human tau, either locally within the temporal region where both transgenes were most active, or in distant areas that were synaptically connected to the cortical region where amyloid deposits initially appeared.

## Materials and Methods

### Mice

**Fig 1:**
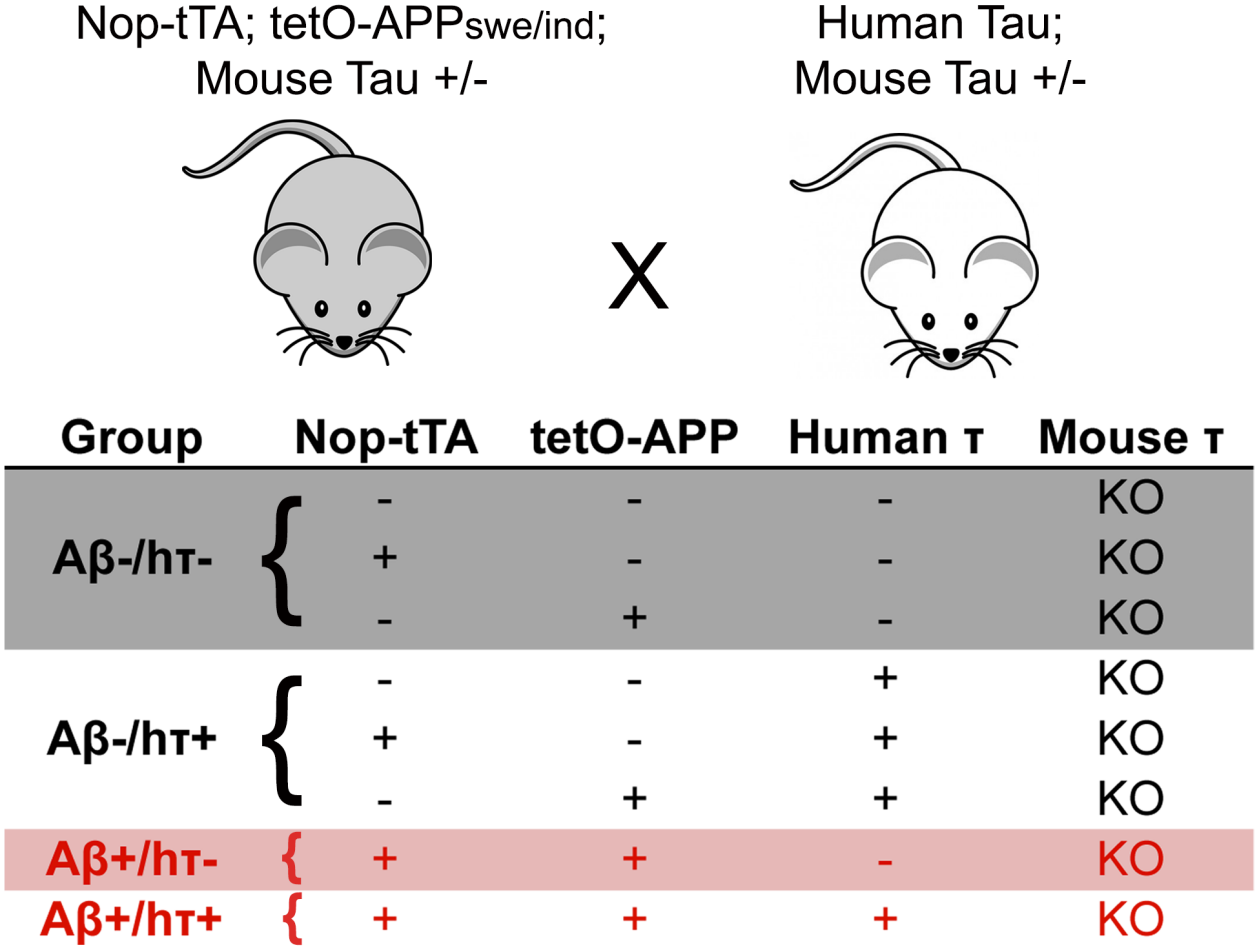
Breeding scheme used to express APP_swe/ind_ on a humanized tau background. *MAPT* hemizygous animals carrying complementary Nop-tTA, tetO-APP, and htau transgenes were interbred to produce animals for this study. The resulting 8 genotypes of *MAPT* null mice were combined into 4 phenotypic groups based on the presence or absence of Aβ and human tau.

Mice for these studies were generated by intercrossing three transgenic lines (Nop-tTA, tetO-APP, and htau) onto the targeted *MAPT* deletion. Our studies used only mice that were null for endogenous tau, but included all 8 possible transgene combinations on the null background. For the sake of clarity, we have simplified the analyses into 4 groups from these 8 genotypes: 1) mice without human tau or transgenic APP [abbreviated here as Aβ-/htau-], 2) mice expressing human tau but not transgenic APP [Aβ-/htau+], 3) mice expressing transgenic APP but not tau [Aβ+/htau-], and 4) mice that expressed both human tau and transgenic APP [Aβ+/htau+]. These groups are illustrated in Fig 1 and included:

1. mice that were entirely non-transgenic, or were singly transgenic for either Nop-tTA or tetO-APP
2. mice that were htau transgenic, but could be non-transgenic or singly transgenic for either Nop-tTA or tetO-APP
3. mice that were transgenic for both Nop-tTA and tetO-APP and non-transgenic for htau
4. mice that carried all three transgenes

There were no statistically significant differences between genotypes within each category for any of the metrics tested. All animal procedures were in accordance with the National Institutes of Health Guide for the Care and Use of Laboratory Animals, and approved by the Baylor College of Medicine Institutional Animal Care and Use Committee under protocol # AN-4975.

#### Neuropsin (Nop)-tTA mice

Mice expressing the tetracycline-transactivator under control of the mouse neuropsin (Nop) / protease serine 19 (Prss19) / kallikrein-related peptidase 8 (Klk8) promoter were generously provided by Dr. Mark Mayford (Line tTA-EC[18], Mutant Mouse Resource and Research Centers (MMRRC) stock #031779-MU). The transactivator expressed in this line had been optimized into human codon usage, and is a variant of the published tTA2^S^ sequence [20]. The animals carry a BAC transgene engineered to place the tTA coding sequence within the first exon of the neuropsin gene, upstream of the translation initiation site. Yasuda and Mayford selected the transgenic founder displaying greatest specificity within the entorhinal cortex (originally known as Line S). The line was backcrossed to C57BL/6 for at least 3 generations prior to the time we received them in 2010. We then backcrossed the line to C57BL/6J for an additional 1–3 generations before outcrossing to the tetO-APP and htau/mtau lines described below.

#### tetO-APPswe/ind mice

Mice carrying a chimeric mouse/human amyloid precursor protein (APP) containing the Swedish and Indiana familial AD mutations under the control of the tetO operator sequence were developed as a conditional, tet-responsive model [17]. We chose line 8–85 for this study because it displays the highest level of transgene expression of the tetO-APP lines that were originally characterized (MMRRC stock #034844-JAX). TetO-APP mice were generated on a hybrid B6C3 F2 background, and backcrossed to C57BL/6J for >20 generations before being outcrossed to Nop-tTA for these studies.

#### Humanized microtubule associated protein tau (htau;mtau KO) mice

Mice expressing the wild-type human microtubule associated protein tau (htau transgenic line 8c) on a mouse tau null background were originally described by Andorfer et al. ([16]; Jackson Laboratory stock #5491). The htau transgene was derived from a human PAC library and retains the endogenous control elements for expression of human tau mRNA [14]. The targeted mouse *MAPT* disruption was created by insertion of GFP into exon 1 of the MAPT locus, resulting in the expression of GFP fused to the first 31 aa of mouse tau protein [15]. The two models were interbred to create a humanized tau mouse, which we received as heterozygous founders from the laboratory of Dr. Hui Zheng. The line had been backcrossed for at least 6 generations onto a C57BL/6J background before we received it, and we carried the backcross for another two generations before outcrossing to tTA/APP transgenic lines for these experiments.

#### Final breeding scheme

Because this experiment involved four independently assorting alleles with a 1/16 chance of generating triple transgenic offspring that were also MAPT null, we elected to outcross all of the lines to an outbred ICR strain background that would produce large litters and thereby decrease the time required to collect the needed cohorts. We began by mating bigenic Nop-tTA/tetO-APP males from a congenic B6 background to wild-type ICR females. Offspring from this cross were then bred to htau; mtau KO animals that had been maintained on a B6 background. Finally, the mtau heterozygous offspring from this cross, each carrying some combination of the Nop-tTA, tetO-APP, and htau transgenes, were interbred with mates carrying a complementary set of transgenes to produce mice for these experiments (see Fig 1).

### Behavioral analyses

Behavioral testing began at 12–13 months of age and included open field (OF), Morris water maze (MWM), radial arm water maze (RAWM), and contextual fear conditioning (CFC). The testing protocol was originally developed for analysis of cognitive behavior in CaMKIIα-TTA/tetO-APP mice as described in Fowler et al. [21] and is copied below for reference. Animals were handled for 3 d before the start of behavioral testing. Locomotor activity in the open field was assessed on day 1, followed by MWM training on days 2–14, and RAWM training on day 15. Mice were allowed a 2 d rest before fear conditioning training on day 18, followed by a retention test on day 19.

#### Open-field assessment (OF)

Locomotor activity was assessed in white acrylic open-top boxes (46 x 46 x 38 cm) in a room lit by indirect white light. Activity was recorded for 30 min and analyzed using the ANY-maze Video Tracking System (Stoelting).

#### Morris water maze (MWM)

MWM testing was conducted in a circular tank measuring 58 cm high and 122 cm in diameter. The water level was 20 cm from the top of the tank and made opaque using nontoxic white paint. Water temperature was maintained between 21 and 23°C. The room was lit with indirect white light and trials were recorded and tracked using ANY-maze Video Tracking System. Before the start of acquisition training, mice received 1 d of training in a straight swim channel to acclimate them to the water and check for motor deficits. Mice received eight trials with a 15 min intertrial interval (ITI) in a channel constructed of white acrylic and measuring 107 x 56 x 14 cm, which was placed in the center of pool. Visible cues were removed from the room during straight swim shaping trials. Mice were allowed 60 s to reach a submerged platform on the opposite side of the channel. Mice that failed to reach the platform were guided to the location by the experimenter. Mice were allowed to stay on the platform for 15 s before being removed from the water, dried, and returned to their home cage under an infrared lamp between trials.

Acquisition training in the MWM consisted of four trials per day with a 15 min ITI. Each training session ended with a short-term memory probe. A square platform (10 x 10 cm) covered in nylon mesh for traction was located 1 cm below the surface in the NE quadrant of the maze, half way between the side and the center of the pool. Mice were placed in the maze facing the wall at each of four cardinal start locations and allowed 60 s to locate the hidden platform. As with straight swim, animals that failed to locate the platform in the allotted time were gently guided there by the experimenter. Mice were allowed to stay on the platform for 15 s before being returned to their home cage between trials. Following the 4 training trials, the platform was removed from the maze for an immediate probe trial. Animals were placed in the tank half way between the cardinal points (SW, NW, SE, and NE) and allowed 45 s to navigate the maze. Proximity to target, percentage time, and percentage path spent in each quadrant were calculated along with the number of times the animal crossed the platform location compared with the other three potential platform locations (in the SW, NW, and SE quadrants). Mice were trained to a performance criterion during probe trials of 35% path in the correct quadrant and at least twice as many of total platform crossings over the correct site compared with any given other possible platform location. When mice reached criterion, they were retired from further MWM training. Mice were trained for a maximum of 12 d. After all mice either reached criterion or completed 12 d of training, each was given one additional “refresher” day of training (four trials) with probe test to ensure equivalent performance between groups before starting RAWM training.

#### Radial arm water maze (RAWM)

The day following MWM refresher training, mice received 1 d of RAWM training consisting of eight trials with a 15 min ITI. The RAWM was created by installing clear Plexiglas triangular inserts into the existing water maze pool (41.25 cm on each side x 50 cm high), which resulted in six open arms joined at the center. Each arm measured 20 cm wide x 34 cm long, and the water was maintained at a depth of 38 cm. The platform was located 3 cm from the end of one arm and submerged 1 cm below the water’s surface. Mice were placed into a different arm at the beginning of each trial, with the order of starting positions pseudorandomly selected before training such that no trial began in an arm adjacent to the previous start position. Mice were allowed 60 s to navigate the maze. If a mouse failed to locate the platform in the allotted time, it was gently guided to there by the experimenter and allowed to remain on the platform for 15 s before being returned to its home cage between trials. Latency to locate the platform, swim path length, and number of working memory errors (re-entries into a previously visited arm) were calculated for each trial. Trial 1 scores were excluded from analysis due to the inflated error rate as animals learned the new procedure. Data from trials 2–8 was added together to provide a performance index for overall RAWM learning.

#### Contextual fear conditioning (CFC)

Two days after RAWM testing, mice were trained in CFC using a near-infrared video fear conditioning system (Med Associates). Conditioning boxes (30.5 x 24 x 21 cm) with a stainless steel grid floor were located inside sound-attenuating chambers and indirectly lit from above. Movement was recorded by a video camera mounted inside the sound attenuating chamber and analyzed using Video Freeze software (Med Associates). Motion threshold was set to 19 arbitrary units (a.u.) and the minimum freeze duration to 1 s. Chambers and grid floors were cleaned with a 20% ethanol solution after each trial. During conditioning, mice were allowed to freely explore the chambers for 2 min before receiving a 1 s, 0.8 mA foot shock. After a 2 min interval, mice received a second 1 s, 0.8 mA footshock. Mice remained in the chamber for 1 min after second footshock before being returned to their home cage. Twenty-four hours later, animals were returned to the same conditioning box for a 5 min retention test. The duration of immobility was recorded and used as an index of learning.

### Tissue harvest

Animals were killed by anesthetic overdose with sodium pentobarbital (Fatal-Plus), followed by thoracotomy prior to transcardial perfusion with ice-cold phosphate-buffered saline (PBS) containing 10 U/ml of heparin. The brain was extracted and bisected along the midline. The caudal third of the right cerebral cortex (containing entorhinal and visual cortices) was collected and frozen on dry ice. This tissue was stored at -80°C for subsequent biochemical assessment. The left hemisphere was post-fixed by immersion in 4% PFA/1x PBS at 4°C for 24 h and was then cryoprotected by immersion in 30% sucrose/1x PBS until it sank. This hemisphere was then sectioned at 35 μm in the horizontal plane on a freezing sliding microtome. Sections were stored in cryoprotectant (0.1M phosphate buffer pH 7.4, 30% ethylene glycol, 25% glycerol) at -20°C until use.

### Histology

#### Campbell—Switzer silver stain

A detailed protocol for this stain can be found online at the NeuroScience Associates website: https://www.neuroscienceassociates.com/reference/papers/alzheimers-disease-pathology-silver-stain

### Amyloid quantitation

Two ventral sections spaced at 280 um apart were stained using the Campbell-Switzer silver method for amyloid detection. The sections were located at interaural 2.36 mm/Plate 148 and interaural 2.64 mm/Plate 150 of the mouse brain atlas by Franklin and Paxinos [22]. Tissue from all Aβ+ animals was stained in a single batch and imaged under the same exposure conditions. Brightfield photomicrographs of each section were taken to cover the caudal cortex and hippocampal formation, and were converted to greyscale using ImageJ64 software. The region of interest (ROI) was defined with reference to the Franklin and Paxinos atlas to include the caudomedial and medial entorhinal cortices plus the neighboring pre- and parasubiculum. Signal was separated from background using the automated threshold function in ImageJ64 to calculate the amyloid percent area used for analysis.

#### Tau immunohistochemistry

Sections were rinsed in tris-buffered saline (TBS), treated with 0.6% hydrogen peroxide in TBS containing 0.1% Triton-X (TBST) for 20 min, and washed again before undergoing antigen retrieval in 10 mM sodium citrate pH 6.0 for 30 min at 80°C. Non-specific binding was blocked by 90 min incubation in TBST containing 5% normal goat serum, before sections were transferred to primary antibody diluted 1:500 in blocking solution for overnight incubation at 4°C (mouse anti-phospho serine 202/threonine 205 tau antibody (AT8, Life Technologies, MN1020) or conformation specific mouse anti-tau (MC1, made by Dr. Peter Davies). Sections were rinsed in TBS before being incubated in biotinylated goat anti-rabbit secondary antibody (Vector Laboratories, BA-1000) diluted 1:500 in blocking solution. After several rinses in TBS, tissue was incubated for 30 min at room temperature with HRP-avidin conjugate diluted 1:50 in TBS. Sections were developed with DAB (D4418, Sigma), then mounted, dehydrated, and coverslipped with Permount.

### Immunoblotting

#### Amyloid precursor protein (APP)

Frozen samples from caudal cortex were homogenized by sonication in 2% SDS containing a protease inhibitor (Sigma # 5892970001)/phosphatase inhibitor (Sigma #4906845001) cocktail and then diluted 1:1 with a modified RIPA buffer (2x PBS, 2% SDS, 1% deoxycholate, 1% NP40, and 5 mM EDTA). Approximately 50 μg of protein was separated on a 4–15% Tris-HCl gel (Bio-Rad, Criterion) and transferred to nitrocellulose (Bio-Rad Trans-Blot Turbo **#**1704155). Membranes were blocked in TBS containing 0.1% Tween 20 (TBSTw) and 5% nonfat dry milk for 2 h at room temperature, and then divided into two pieces prior to addition of primary antibody. The upper half of the blot was incubated with human-specific APP/Aβ antibody 6E10 (1:5000; BioLegend #803015) and total APP antibody Y188 (1:2500; Abcam #ab32136); the lower half of the blot was incubated with GAPDH antibody (1:10000; Millipore #AB2302) as a loading control. After several washes in TBSTw, blots were incubated for 2 h at RT with dye-conjugated secondary antibodies (IRDye 680RD donkey anti-rabbit #926–28073; IRDye 800RD donkey anti-mouse #827–08364; or IRDye 680RD donkey anti-chicken #926–68075; all from Li-Cor; each diluted 1:10,000). Binding was captured using the Li-Cor Odyssey Fc imaging system and quantified using Image Studio software.

#### Total and phosphorylated human tau

The remaining frozen tissue samples were mechanically homogenized on ice using a Polytron tissue grinder in five volumes of TBS containing 10% sucrose plus protease inhibitor (Sigma # 5892970001) and phosphatase inhibitor cocktails (Sigma #4906845001). This homogenate was centrifuged at 800 x g for 5 min in a refrigerated microcentrifuge. The resulting pellet was resuspended in an equal volume of the same solution and again centrifuged at 800 x g for 5 min. The two supernatants were combined before being centrifuged at 100,000 x g for 1 h at 4°C. The high-speed supernatant was collected and saved as the soluble fraction. The high-speed pellet was resuspended in TBS containing 1% sarkosyl plus protease/phosphatase inhibitor and incubated on a rotating platform for 1 h at RT. Following this incubation, the resuspended pellet was centrifuged at 100,000 x g for 1 h at 4°C. The resulting high-speed pellet was resuspended in TBS containing 1% SDS and protease/phosphatase inhibitor, sonicated, and saved as the sarkosyl-insoluble fraction. Approximately 30 μg of each fraction was separated on 4–15% Tris-HCl gels (BioRad Criterion) and transferred to nitrocellulose as above. Membranes were then incubated with a combination of 3 primary antibodies (GAPDH + two anti-tau antibodies) diluted in blocking solution (TBSTw containing 5% nonfat dry milk). The following total or phospho-specific tau antibodies were used: total human tau (rabbit anti-human tau; 1:1000; #A0024; DAKO), AT8 (mouse anti-phospho-tau, serine 202/threonine 205; 1:2500; #MN-1020; Pierce Antibodies), AT270 (mouse anti-phospho-tau, threonine 181; 1:500; #MN-1050; Pierce Antibodies), and PHF1 (mouse anti-phospho-tau, serine 394/404; 1:2500; made by Dr. Peter Davies). GAPDH was used as a loading control. Binding was detected with dye-conjugated secondary antibodies and imaged with a Li-Cor Odyssey machine as described above.

### Statistics

Statistical comparisons between two groups were made using a Student’s t-test, and between three or more groups using one-way or two-way ANOVA, as appropriate. All *post hoc* comparisons were conducted using Bonferroni correction. Prism 6.0 (GraphPad) was used for all statistical analyses. Graphs display group mean ± SEM and significance values indicated in figures are for *post-hoc* comparisons. Figures use standard nomenclature for indicating significance levels: * *p* < 0.05, ** *p* < 0.01, *** *p* < 0.001, **** *p* < 0.0001.

## Results

### Creating a model with regionalized amyloid and widespread human tau expression

Mouse models designed to test the amyloid cascade hypothesis have generally combined wild-type human Aβ, either by injection or by transgenesis, with a human tau variant identified from FTLD [9–13]. However, tau mutations have yet to be associated with familial Alzheimer’s disease [3], making the use of aggregation-prone variants from other tauopathies a less-than-ideal test of the hypothesis. Our goal in this study was to test if Aβ could exacerbate tauopathy in a mouse model expressing the same tau isoforms as an AD patient. We used a model developed by Duff and Davies in which the wild-type human tau genomic locus was expressed mice lacking the endogenous tau gene [16]. To maximize our chances of seeing an interaction with Aβ, we chose to cross this human tau model with an aggressive double-mutant APP model [17, 19]. We further used a transgenic driver line that placed the highest levels of Aβ expression within the entorhinal region that shows the earliest and most severe degeneration in AD patients [23, 24].

Because the crosses to produce this bipartite tau-Aβ model generated 24 distinct genotypes, we elected to simplify our analysis by focusing on offspring that were null for mouse tau. Even with this selection, the combination of three transgenes still resulted in eight distinct genotypes that were included for study. These were further consolidated into four experimental groups representing the primary comparisons intended for this study: 1) negative for both amyloid and htau, 2) positive for htau but negative for amyloid, 3) positive for amyloid but negative for htau, or 4) positive for both amyloid and htau (Fig 1). Each amyloid-negative group was composed of three genotypes that were initially analyzed separately, but later combined after confirming that there were no significant differences among them (data not shown). Mice were behaviorally tested at 12–13 months of age, and were harvested at 13–14 months of age for biochemical and histological analysis. We selected this age for study based on past characterization of both models. The humanized tau model is meant to have mild tau hyperphosphorylation by this age, while the neuropsin-APP mice have been shown to harbor moderate amyloid pathology [16, 19].

### Wild-type human tau and pathogenic APP have independent effects on locomotor activity and memory

We began our analysis of the new htau-amyloid model with cognitive testing. Although the behavioral impact of mutant APP has been well-characterized, few papers have explored the potential interaction with wild-type human tau.

**Fig 2:**
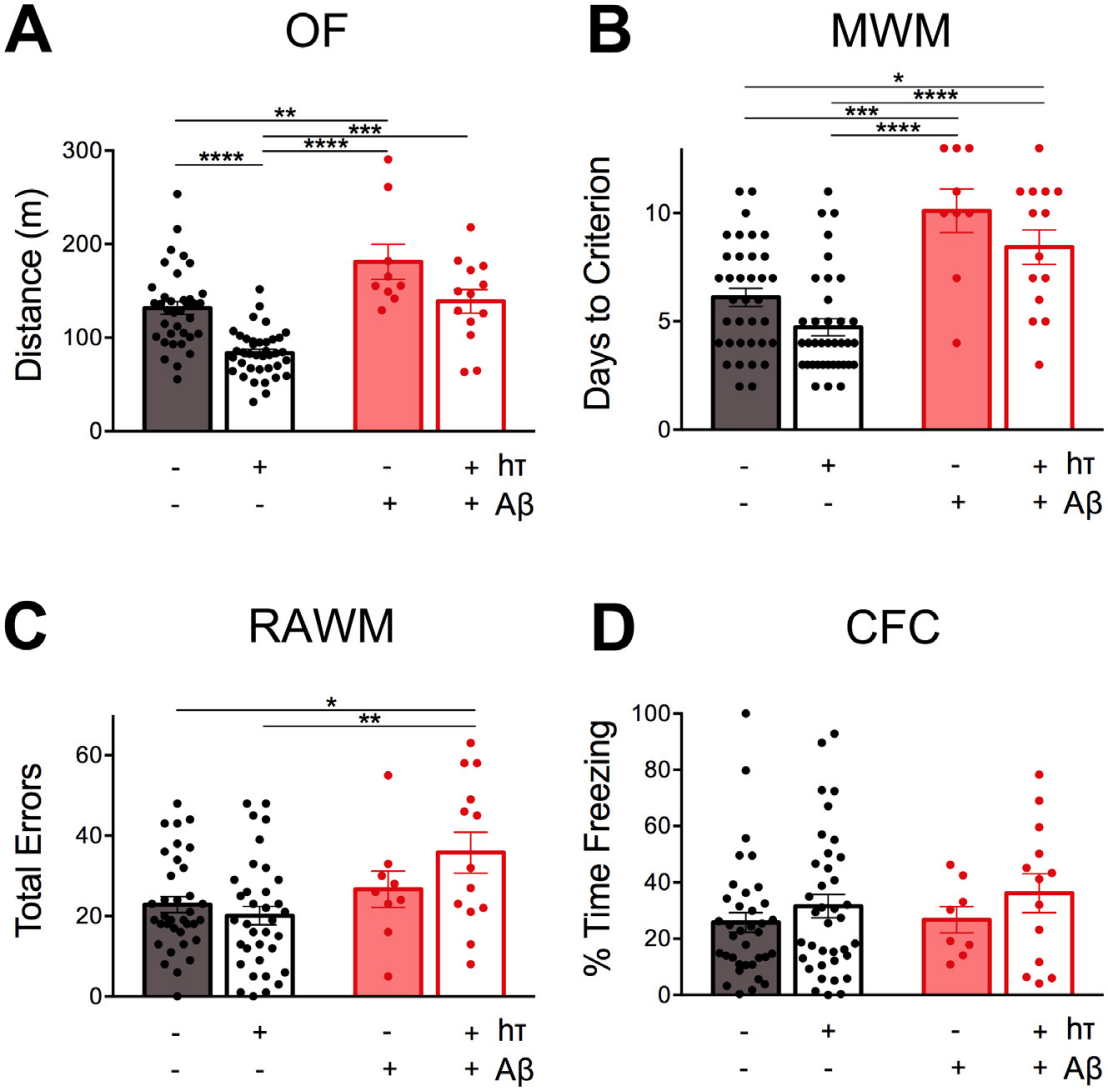
Transgenic APP and htau have independent behavioral effects in aged mice. A. Distance traveled in the open field assay (OF). B. Days to criteria performance in the Morris water maze (MWM). C. Total arm re-entry errors in the radial arm water maze (RAWM). D. Percent of time freezing in contextual fear conditioning (CFC). Individual values are plotted for each group, amyloid-bearing mice in red, amyloid-free mice in black. Mice expressing htau are shown with open bars, mice without htau are shown with solid bars.

Our behavioral analyses always begin with a gross assessment of locomotor performance using the open field assay. We noted significant effects of both Aβ and human tau on distance traveled in the open field (two-way ANOVA, F_(1,92)_ = 31.34, *p* < 0.0001 for Aβ; F_(1,92)_ = 23.30, *p* < 0.0001 for htau; F_(1,92)_ = 0.09704, *p* = 0.7561 for interaction; Fig 2A). Mice overexpressing Aβ but not htau (Aβ+/htau-) traveled the greatest distance, while Aβ-/htau+ mice traveled the least (Fig 2A). This latter group of mice, expressing transgenic htau but not Aβ, traveled significantly less than all three other groups. Conversely, the absence of htau revealed a significant effect of Aβ, with htau-/Aβ+ mice traveling greater distances than htau-/Aβ- mice. Despite the effect of genotype on distance, we found no differences in center/perimeter occupancy, either by time or distance (data not shown).

We next tested spatial learning and memory using the Morris water maze (MWM). Pre-training in a straight swim channel revealed no impact of genotype on swim speed (data not shown), suggesting that the differences in open field behavior did not affect performance in the water. We conducted a modified version of the MWM in which animals are trained on successive days until they reach pre-set performance criteria that are evaluated during probe trials immediately after training. This approach revealed that entorhinal expression of transgenic Aβ significantly delayed acquisition of the task (two-way ANOVA, F_(1,92)_ = 37.72, *p* < 0.0001 for Aβ; F_(1,92)_ = 0.05771, *p* = 0.8107 for interaction; Fig 2B). Aβ+/htau- mice required the greatest number of days to reach criterion performance, with Aβ+/htau+ mice coming in a close second. Unexpectedly, the presence of human tau significantly improved acquisition of this task (two-way ANOVA, F_(1,92)_ = 5.973, *p* < 0.0001 for htau), although this effect was not robust enough to yield significant post-hoc comparisons.

Following completion of MWM, mice underwent testing in the radial arm water maze (RAWM). This task examines working memory during acquisition of a modified water maze based on navigation of a 6-arm swimming arena. Re-entry into an arm that has already been explored during that trial is counted as an error, and scores are assessed from total errors made during trials 2–8. Aβ+/htau+ mice performed significantly worse than both non-amyloidogenic groups, (Fig 2C). This impairment was not seen in Aβ+/htau- mice, suggesting that the working memory deficit seen in mice with both Aβ and htau is due to an interaction between them. This interaction is suggested, but not substantiated, by the near-significant statistical interaction between these factors: two-way ANOVA, F_(1,88)_ = 8.251, *p* = 0.0051 for Aβ; F_(1,88)_ = 0.8809, *p* = 0.3505 for htau; F_(1,88)_ = 3.056, *p* = 0.0839 for interaction.

Animals were given several days to recover before being tested in contextual fear conditioning (CFC) to examine associative memory. All groups showed the same level of baseline freezing prior to receiving the first shock on training day (data not shown). Unexpectedly, all groups also showed the same level of conditioned fear during the context test (Fig 2D). This was especially surprising because CFC is known to be hippocampal-dependent, and we expected that amyloid pathology in the entorhinal region might influence acquisition or consolidation of contextual information.

In summary, our first round of behavioral tests revealed that the regionalized overexpression of mutant APP and the generalized expression of wild-type human tau had significant but independent effects on locomotion, spatial learning, and working memory.

### Wild-type human tau normalizes behavioral alterations in MAPT null mice

**Fig 3:**
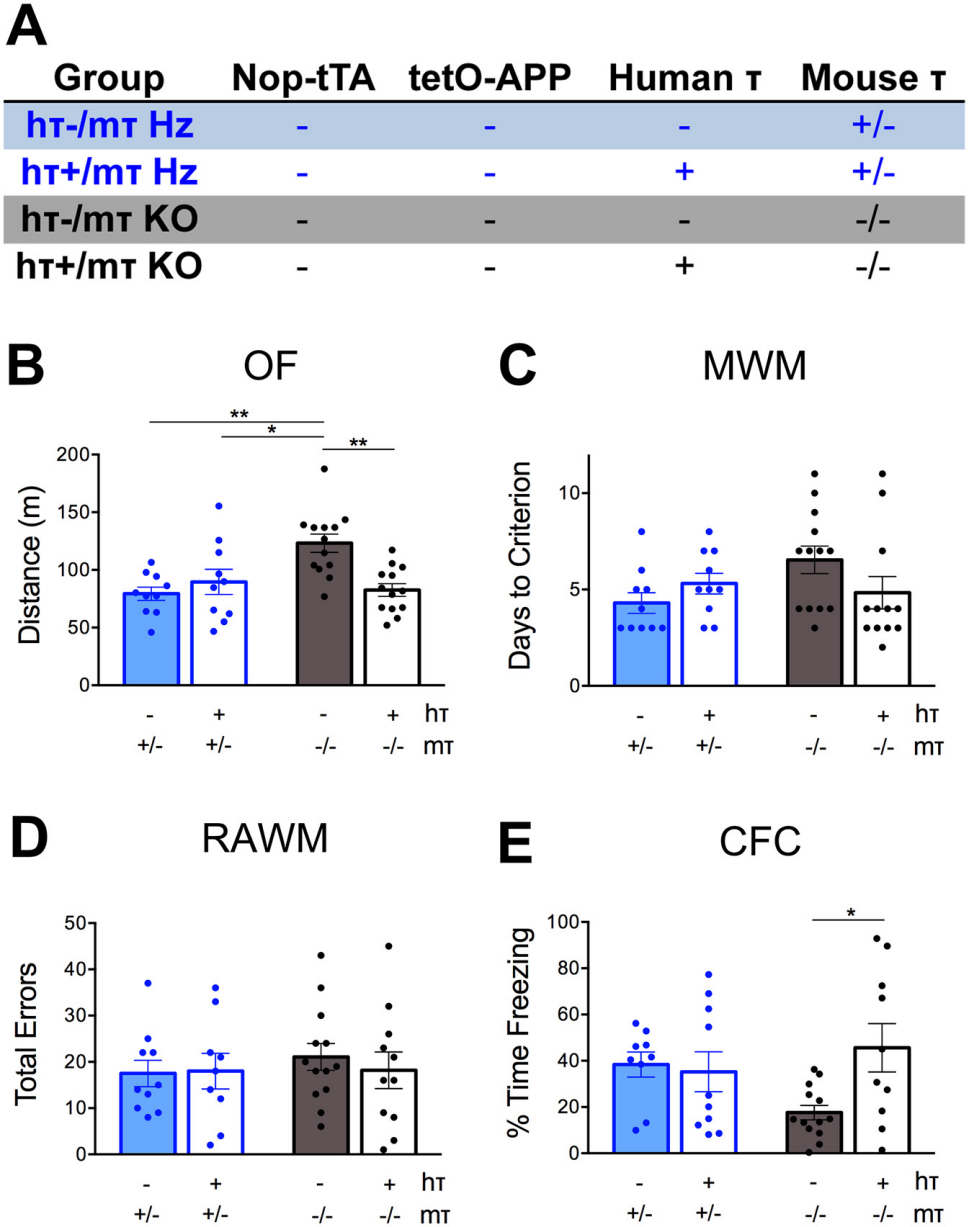
Behavioral changes caused by loss of endogenous tau are rescued by transgenic expression of wild-type human tau. A. The breeding scheme described in Fig 1 also generated *MAPT* hemizygous mice that were later added to behavioral testing. *MAPT* hemizygous animals were compared with their *MAPT* null littermates to distinguish whether behavioral phenotypes observed in Aβ/htau mice were due to the presence of human tau or to the loss of mouse tau. Mice lacking any form of tau exhibit hyperactivity in open field (OF; panel B) and impaired freezing in contextual fear conditioning (CFC) tasks (panel E). In each case, this effect is ameliorated by the addition of wild-type human tau. No effect of tau genotype was observed in MWM (panel C) or RAWM (panel D). Individual values are plotted for each group, *MAPT* hemizygotes in blue, *MAPT* null mice in black. Mice expressing htau are shown with open bars, mice without htau are shown with solid bars.

As we completed behavioral testing of the Aβ/htau mice, our ongoing data analysis lead us to question whether the absolute level of tau protein—rather than the species from which it derived—was affecting some aspects of behavior. We were especially struck by three observations: 1) the significant hypoactivity of Aβ-/htau+ mice in OF, 2) a main effect showing faster MWM acquisition in htau+ groups, and 3) the relatively poor CFC performance by all groups. We suspected that in some of these instances, expression of htau was attenuating phenotypes caused by loss of mouse tau. To explore this possibility, we began to include sibling *MAPT* hemizygous mice in our testing that were produced from our breeding scheme. We compared performance of these *MAPT* hemizygous animals to a subset of the *MAPT* null mice that also lacked both tTA and APP. Using these mice, we could directly evaluate the effect of tau copy number (0 vs. 1) and species (mouse vs. human) (Fig 3A).

Analysis of OF activity revealed a significant effect of tau level on distance traveled (two-way ANOVA, F_(1,42)_ = 3.882, *p* = 0.0554 for htau; F_(1,42)_ = 5.677, *p* = 0.0218 for mtau; F_(1,42)_ = 10.96, *p* = 0.0019 for interaction; Fig 3B). Mice lacking any form of tau protein were significantly hyperactive compared to all other genotypes. Expression of one copy of either htau or mtau abated this hyperactivity, with no further change observed in mice expressing both htau and mtau.

Our initial studies in *MAPT* null mice indicated a main effect of htau on spatial learning in the MWM (Fig 2B). However, we now found no further effect of mouse tau on the number of days required to reach criteria performance (Fig 3C). Unlike MWM, performance in the RAWM test of working memory was not affected by tau levels (Fig 3D). There were no significant differences between mtau and htau genotypes on RAWM, consistent with our initial experiment in which only mice carrying both htau and Aβ displayed impairment (Fig 2C).

Analysis of associative memory in CFC was complicated by the relatively poor performance in all four groups during our initial testing of *MAPT* null mice (Fig 2D). When hemizygous *MAPT* mice were now included in the analysis, we observed a significant interaction between human and mouse tau for the percent time freezing during context testing (two-way ANOVA, F_(1,38)_ = 2.977, *p* = 0.0926 for htau; F_(1,38)_ = 0.535, *p* = 0.469 for mtau; F_(1,38)_ = 4.69, *p* = 0.0367 for interaction). Expression of htau abated the impairment caused by loss of mouse tau, but had no further impact in hemizygous animals (Fig 3B).

Taken together, data from this second round of behavioral analysis suggest that removing mouse tau from the brain can significantly impact both locomotion and associative learning/memory. The presence of either wild-type human tau or a single copy of mouse tau attenuated these phenotypes, arguing that some minimum level of either human or mouse tau is sufficient to restore normal behavior in these tasks.

### APP expression and amyloid pathology are unaffected by wild-type human tau

**Fig 4:**
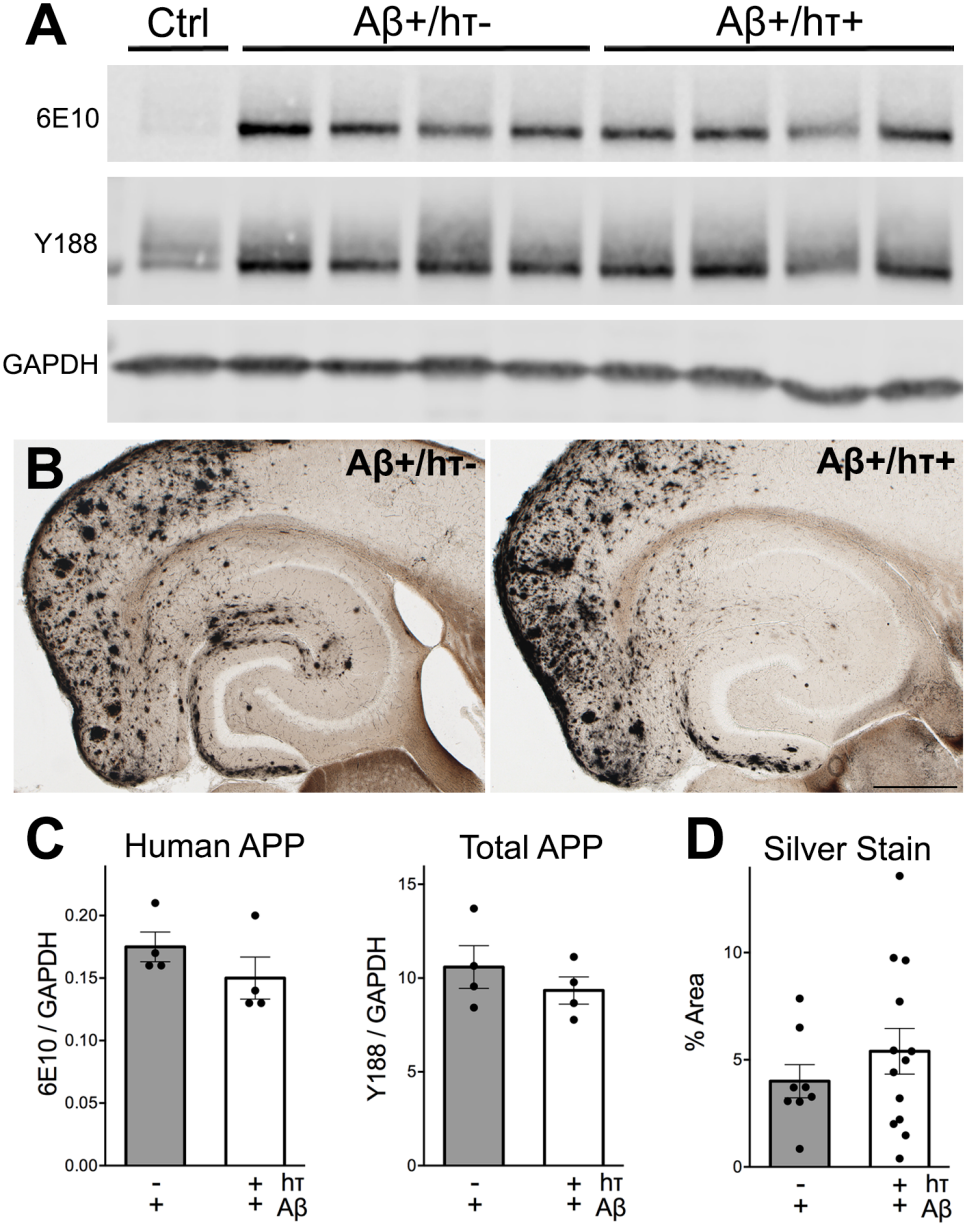
Human tau does not affect the expression of transgenic APP or the severity of amyloid pathology. A. Western blots of cortical homogenate were probed with 6E10 and Y188 to measure the expression of transgenic and total APP. B. Campbell-Switzer silver stain was used to detect amyloid pathology in Aβ+ mice with and without htau. Scale bar, 500 μm. C. Semi-quantitative analysis of 6E10 and Y188 Western blots shown in A. D. Image analysis of silver stains shown in B, displayed as percent area occupied by amyloid staining.

After behavioral testing was complete, all mice were harvested so that we could examine the biochemical and histological interactions between htau and Aβ. We first tested whether the presence of human tau had any effect on the expression of transgenic APP or the resulting amyloid pathology in entorhinal cortex of neuropsin-APP mice with or without the htau transgene. We chose a random subset of APP/TTA animals evenly divided between male and female to examine transgene expression in the entorhinal region. Consistent with past studies [25–27], we found no change in the steady-state level of APP when probed with either the human-specific antibody 6E10 or the species-independent antibody Y188 (Fig 4A and 4C). We next examined the severity of amyloid pathology in the entorhinal area using silver stain for detection. With or without htau, both groups of Aβ+ mice developed robust amyloid deposits. Neither the spatial pattern nor the area of amyloid staining differed between groups (Fig 4B and 4D). These findings suggest that the expression of human tau has no overt effect on the production of endogenous or transgenic APP or the resulting amyloid deposits that arise from human Aβ production.

### Aβ aggregation does not significantly alter phosphorylation of wild-type human tau

**Fig 5:**
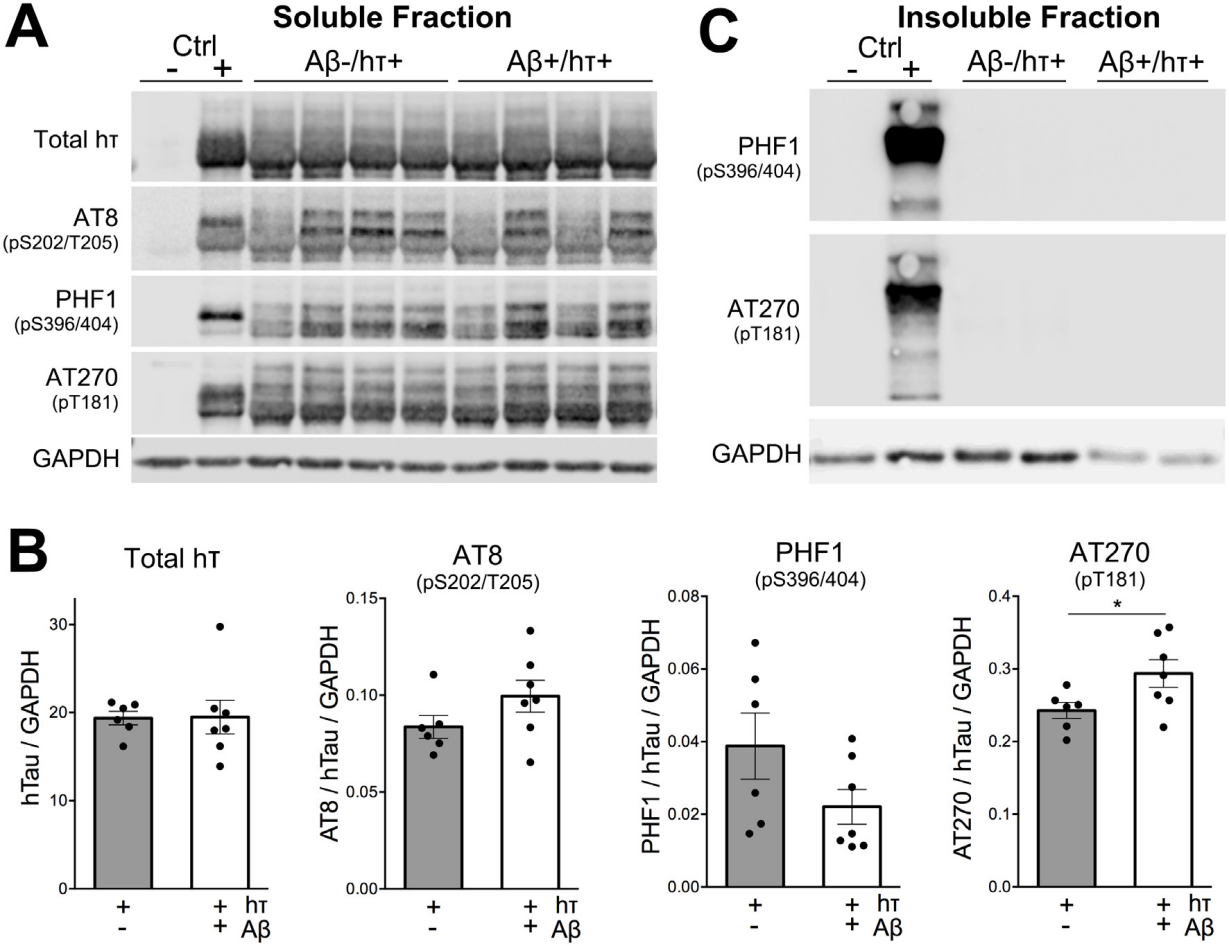
Regionalized amyloid pathology has a negligible effect on the levels or phosphorylation of wild-type human tau. Levels of total and phospho-tau were assessed by Western blot using AT8, AT270, and PHF1 antibodies. A. Representative Western blots of the soluble fraction from cortical homogenates. B. Semi-quantitative measurement of signal intensity from immunoblots for each phospho-epitope. Only phospho-threonine 181 showed a slight increase in the presence of amyloid. C. No sarkosyl-insoluble tau was detected in htau+ mice at this age with any antibody tested. Blots probed with PHF1 and AT270 are shown as representative examples. Homogenate prepared from a 5 month old rTg4510 mouse served as a positive control tissue for all blots.

We next examined whether Aβ aggregation, driven by the overexpression of mutant APP, would be sufficient to accelerate the hyper-phosphorylation of wild-type human tau. We tested a series of phospho-specific tau epitopes, both biochemically on Western blot and histologically in fixed tissue sections. We included tissue from rTg4510 mice expressing high levels of human tau P301L as a positive control for these experiments [7, 28]. rTg4510 mice were harvested at 5 months of age when they harbor substantial tau hyper-phosphorylation and frank neurofibrillary tangles. Our first experiments used a multi-step extraction protocol to separate tau into soluble and sarkosyl-insoluble fractions. Because this extraction differs from the standard RIPA buffer used for APP detection above, our tau analyses focused on the remaining samples. We first examined the extent of tau phosphorylation in the soluble fraction using three well-characterized antibodies: AT8, AT270, and PHF1. We found no difference in the levels of total human tau, and no change in the degree of phosphorylation at serine 202/threonine 205 or serines 396/404. There was a small but significant increase in phosphorylation at threonine 181 in amyloid-bearing htau mice, but altogether, negligible impact of Aβ accumulation on the phosphorylation of cytosolic tau protein (Fig 5A and 5B).

We then examined the same panel of tau antibodies with the sarkosyl-insoluble fraction. While the positive control rTg4510 tissue contained intense signal from all four antibodies, neither group of htau animals displayed any fibrillar tau. The results from two of the antibodies, PHF1 and AT270, are shown in Fig 5C.

**Fig 6:**
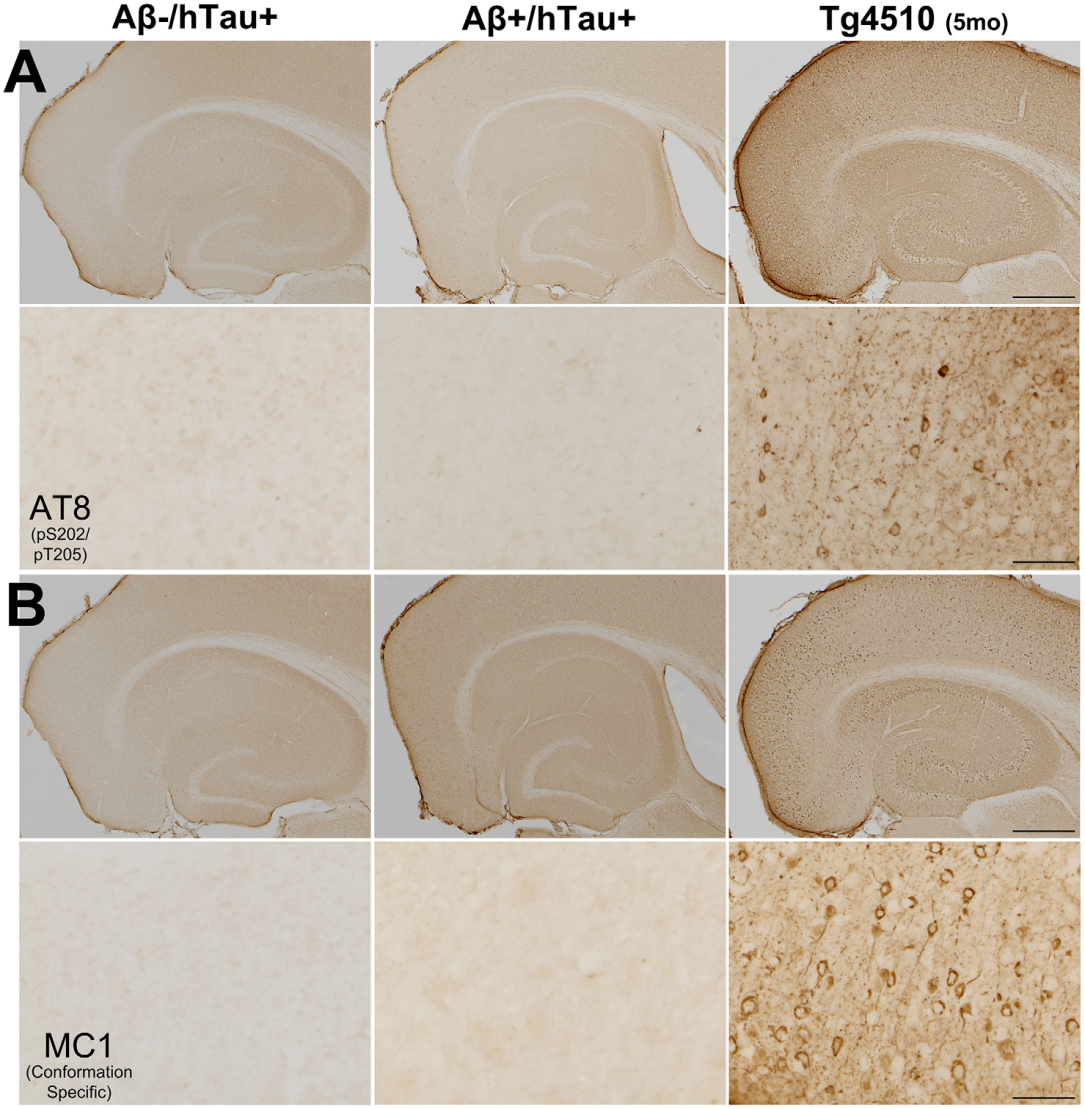
Absence of intra-neuronal tau accumulation in htau mice regardless of amyloid genotype. A. Immunostaining for AT8 was clearly visible in rTg4510 mice, but absent from htau+ mice. B. MC1 immunostaining again shows tau aggregates in rTg4510 mice, but is absent from htau+ mice. Scale bar, 500 μm (top panels), 125 μm (bottom panels).

Despite the lack of insoluble tau on Western blots, we were optimistic that immunohistochemistry on fixed tissue sections might provide a more sensitive means of detecting hyper-phosphorylated tau described in the humanized tau model [16]. We selected two tau antibodies that showed particularly strong signal in the positive control rTg4510 mice: AT8 and MC1. Despite robust signal in the rTg4510 tissue, we detected no immunostaining with either antibody in htau transgenic animals regardless of their amyloid status (Fig 6A and 6B).

## Discussion

Research in Alzheimer’s disease (AD) has sought a mechanistic link between Aβ and tau since the amyloid cascade hypothesis was first proposed nearly 25 years ago [1]. Previous studies have demonstrated that both exogenous Aβ infused through a needle and intrinsic Aβ derived from transgenic APP can exacerbate tauopathy in mouse models of FTLD [9–13]. Here we set out to test whether Aβ could accelerate hyper-phosphorylation and induce neurofibrillary tangles in mice expressing wild-type human tau [16]. Our experiments revealed independent effects of Aβ and htau on locomotor activity, spatial learning, and working memory, but found little evidence of an interaction between the two proteins either behaviorally or biochemically.

Our biochemical and histological analyses focused on the mouse brain regions analogous to the human temporal lobe where neurofibrillary tangles and neurodegeneration first appear in AD [23, 24]. We intentionally chose the neuropsin-TTA driver line for our tet-responsive APP model to concentrate transgenic Aβ in this region to maximize our chances of observing a biochemical interaction with human tau. Consistent with several past studies, we saw no effect of human tau expression on transgenic APP levels or amyloid load [25–27]. Conversely, we found only a small increase in one tau phospho-epitope, with no evidence for tau aggregates or tangle pathology, in mice with robust amyloid pathology. Only a handful of previous studies have examined the impact of Aβ pathology on the phosphorylation of wild-type human tau and they are divided in their findings. The study most similar to ours reached the same conclusion we did. Guo et al. intercrossed the htau mouse with an APP_swe/lon_/PS1_M146V_ knock-in model to create a model in which all three AD-associated human proteins were expressed at endogenous levels in their native spatio-temporal patterns. Despite lifelong Aβ overproduction, APP/PS1/htau animals displayed the same degree of tau phosphorylation as age-matched htau mice at 20 months [27]. Earlier work by Boutajangout et al. tested the effect of transgenic PS1_M146L_ overexpression in the htau model, but in the absence of human APP or amyloid pathology. Immunostaining for both PHF1 and AT8 was stronger in htau/PS1 animals than in htau littermates, with corresponding increases in PHF1 signal on Western blot [29]. Although the mechanism of enhanced tau phosphorylation in the htau/PS1 mice was not explored, the authors suggested the involvement of GSK3β rather than any change in APP processing. Taken together, these studies better support the possibility that Aβ and tau pathologies are distinct processes than interconnected points of a pathogenic cascade [30], however, this view is far from mainstream. Other studies have observed a synergistic interaction between Aβ and wild-type human tau, but in mice carrying just one of the six tau splice variants. Chabrier et al. found that mice co-expressing human APP_swe/lon_ with 2N/4R human tau accumulated more insoluble tau and higher levels of tau phosphorylation than 2N/4R single transgenic siblings [31]. While this model argues in favor of a pathogenic interaction between Aβ and wild-type tau, the absence of 3R isoforms complicates interpretation with regard to AD. We have thus found only one other study directly testing the interaction between Aβ and human 6 isoform wild-type tau in vivo, and it too supports the unexpected conclusion that amyloid may have little bearing on tau pathology.

Our behavioral data provide further evidence that Aβ and wild-type human tau act independently in our model. APP overexpression caused locomotor hyperactivity and impaired spatial learning while human tau expression diminished locomotion but improved spatial learning, yet we found no statistical interaction between genotypes on any of the behavioral tasks. Guo et al. found that the substitution of human tau in place of mouse paradoxically improved recall of contextual fear in APP/PS1 mice without affecting cued fear conditioning, water maze, or open field behavior [27]. Boutajangout et al. similarly found little difference in cognitive performance of htau mice with and without transgenic co-expression of PS1_M146L_ when tested in object recognition or closed field symmetrical maze [29]. Yet in mice carrying the 2N/4R isoform of human tau, Chabrier et al. observed a strong interaction with Aβ in both novel object recognition and water maze [31]. It is difficult to reconcile these conflicting data with our findings. Perhaps of relevance, our study was the only one in which the animals had developed amyloid pathology by the age of cognitive testing; the other models cited were either non-amyloidogenic [29, 31], or were tested prior to amyloid onset [27].

One surprising outcome of our behavioral testing was that removing mouse tau did not abate learning impairments in the neuropsin-APP mice. Roberson et al. had previously reported that learning and memory impairment in APP transgenic mice (line J20) was eliminated by tau deletion [26, 32]. In MWM testing, their APP+/tau- mice performed as well as wild-type controls. Roberson et al. further showed that tau levels alone had no impact on learning or recall in MWM, and our findings support this conclusion. However, in our study Aβ+/tau- mice took significantly longer to learn the MWM than tau null animals (Aβ-/tau-, Fig 2B). This outcome suggests that removing tau is not sufficient to rescue learning impairment caused by transgenic APP/Aβ in our model. Because we did not include Aβ+ mice on a wild-type tau background needed to make a direct comparison of amyloid-associated behavior in the presence and absence of endogenous tau, we cannot draw a definitive conclusion from our data; nevertheless, the significant learning difference with tau null mice strongly predicts this outcome.

While conducting the behavioral testing for this study we discovered that the absolute level of tau expression—regardless of whether it was derived from human or mouse—had a significant influence on locomotor activity. When it became apparent that performance of our htau mice differed from control animals lacking tau, we decided to add littermates hemizygous for mouse tau to our behavioral pipeline. Through this comparison, we found that tau deletion caused locomotor hyperactivity, and with it, impairments in contextual fear memory measured by immobility. Both effects were attenuated by a single copy of mouse tau or by substitution with human tau. Several past studies have documented a progressive locomotor phenotype in various tau knockout mice, although both increased distance and decreased speed/movement in open field activity have been reported and appear to depend on age, gender, and genetic background [33–40]. Locomotor hyperactivity in animals lacking both human and mouse tau may have confounded our ability to detect an interaction between human tau and Aβ by cognitive testing: we may have unintentionally made impairments in neuropsin-APP mice worse by removing mouse tau, and then rescued these tau-deletion deficits with htau. Past studies argue against this possibility, however, with most reporting MWM performance of tau null mice identical to wild-type controls throughout the age range tested here [26, 35, 38–40]. Results are divided with respect to fear conditioning, but half of published reports again suggest no effect of mouse tau genotype on performance [40] (but see [33]). Where hemizgous animals were included, their performance was consistently equivalent to wild-type controls [26, 33, 35, 39, 40]. Taken together, these findings argue that spatial memory function is not affected by the loss of mouse tau, and further suggest that the hemizgous mice included in our experiments offer a reasonable approximation of wild-type performance. Future experiments comparing Aβ+ mice with and without mouse tau will be needed to determine whether this equivalence extends to animals already impaired by amyloid neuropathology.

There are three further caveats to our experimental design that may explain the absence of an interaction between Aβ and tau in our animals—age, genetic background, and amyloid distribution. First, we may simply not have waited long enough for an interaction to occur. We tested mice at 13–14 months of age because htau mice had been reported to develop somatodendritic CP13- and PHF1-positive pre-tangles by this age that would serve as a reference for any change in severity with the addition of Aβ [16, 41, 42]. Although it is possible we might have seen an effect at later ages, Guo et al. examined htau mice up to 20 months of age without observing any change in tau pathology due to Aβ [27]. The Guo et al. findings also diminish the likelihood that regionalized APP expression in our model prevented us from detecting an interaction between tau and Aβ. The APP/PS1 knockin model used by Guo displayed widespread neocortical amyloidosis at late ages, yet they saw no difference in tau markers between htau mice with and without Aβ [27]. The degree of amyloidosis in the APP/PS1 knockin model is substantially lower than attained by APP transgenic mice, and it is possible that had we used a stronger or more ubiquitous tTA driver we might have observed an effect outside of the entorhinal region studied here. Finally, the mixed genetic background of our animals may have masked potential interactions. Although the ICR outbred strain used here is related to the Swiss Webster (SW) strain used in the original derivation of the htau model, the original breeding colonies diverged in the 1930's and now have distinct genetics [43]. Both tau phosphorylation [44, 45] and pathological aggregation [11, 46] can be influenced by strain background in mice. The direction of change is dependent on the model under study: in the JNPL3 model, moving from a hybrid B6/D2/SW to a congenic B6 background substantially delayed the onset of hyperphosphorylation [11], but in the rTg4510 model, the B6;FVB hybrid showed higher levels of phospho-tau at late ages than the original 129;FVB strain [46]. Although it is difficult to compare the onset of pathology across studies, the htau model has shown hyper-phosphorylated, insoluble tau and pre-tangles on a variety of inbred (B6 [27, 41, 47–49] and B6;129 [50]) and outbred strains (SW;129;B6 [16] and SW;B6;129;D2;SJL [29]). Taken together, we predict that age and amyloid distribution rather than strain background will be more productive variables for testing in future studies.

In sum, we find little evidence for a pathological link between Aβ and wild-type human tau in our model. Despite the limitations of our study, we feel that our data are more consistent with a non-linear relationship between amyloid, tau, and dementia than with a direct cascade [3]. Experimental evidence linking Aβ with neurofibrillary tangles has largely relied on mouse models of FTLD and other non-AD tauopathies in which mutant tau protein would ultimately become hyper-phosphorylated even without Aβ [9–13]. The difficulty of producing ‘wild-type tauopathy’ in mice raises important questions about their use in modeling complex neurodegenerative disease. Might rats, non-human primates, or some as yet undetermined model organism with tau isoforms and splicing variants more similar to humans prove a more naturalistic model of the disease [51]? Unlike mice, rats may naturally express all six tau isoforms [52] (but see [53, 54]). Both rats [55–59] and non-human primates [60, 61] have been shown to develop tau pathology upon amyloid exposure. These models are still in their infancy, and mice will continue to be an indispensable tool for neurodegenerative studies because of their low cost, genetic tractability, and short reproductive cycle. Perhaps we are asking the right question in the wrong model, but the evidence so far suggests that we continue to critically re-evaluate our hypothesis at the same time we invest in building better models.
